# The Influence of Cadmium Stress on the Content of Mineral Nutrients and Metal-Binding Proteins in *Arabidopsis halleri*

**DOI:** 10.1007/s11270-012-1292-4

**Published:** 2012-08-23

**Authors:** Ewa Przedpełska-Wąsowicz, Aleksandra Polatajko, Małgorzata Wierzbicka

**Affiliations:** 1 Department of Molecular Plant Physiology, Institute of Botany, Faculty of Biology, University of Warsaw, Miecznikowa 1, 02-096 Warsaw, Poland; 2 ISAS-Institute for Analytical Sciences, P.O. Box 101352, 44013 Dortmund, Germany

**Keywords:** *Arabidopsis halleri*, Cadmium, Cadmium-binding proteins, Hyperaccumulator, Metal accumulation, Zinc

## Abstract

We investigated the influence of cadmium stress on zinc hyperaccumulation, mineral nutrient uptake, and the content of metal-binding proteins in *Arabidopsis halleri*. The experiments were carried out using plants subjected to long-term cadmium exposure (40 days) in the concentrations of 45 and 225 μM Cd^2+^. Inductively coupled plasma-mass spectrometry, size exclusion chromatography coupled with plasma-mass spectrometry, and laser ablation inductively coupled plasma-mass spectrometry used for ablation of polyacylamide gels were employed to assess the content of investigated elements in plants as well as to identify metal-binding proteins. We found that *A. halleri* is able to translocate cadmium to the aerial parts in high amounts (translocation index >1). We showed that Zn content in plants decreased significantly with the increase of cadmium content in the growth medium. Different positive and negative correlations between Cd content and mineral nutrients were evidenced by our study. We identified more than ten low-molecular-weight (<100 kDa) Cd-binding proteins in Cd-treated plants. These proteins are unlikely to be phytochelatins or metallothioneins. We hypothesize that low-molecular-weight Cd-binding proteins can be involved in cadmium resistance in *A. halleri*.

**Electronic supplementary material:**

The online version of this article (doi:10.1007/s11270-012-1292-4) contains supplementary material, which is available to authorized users.

## Introduction

*Arabidopsis halleri* (Brassicaceae) is a perennial species occurring in Europe and East Asia (Al-Shehbaz and O’Kane 2002). Apart from natural mountainous habitats, it occurs also in areas polluted with heavy metals (Ernst 1990; Pauwels et al. 2006). The species is well known for its’ tolerance to zinc and cadmium and the ability to hyperaccumulate these metals. *A. halleri*, a close wild relative of *Arabidopsis thaliana*, is a model species in studies focused on the problem of metal tolerance and hyperaccumulation in plants (Pauwels et al. 2006; Verbruggen et al. 2009; Maestri et al. 2010; Meyer et al. 2010, 2011; Gode et al. 2012).

Many studies have investigated zinc hyperaccumulation in plants (Lasat and Kochian 1998, 2000; Pauwels et al. 2006; Broadley et al. 2007; Verbruggen et al. 2009; Maestri et al. 2010; Meyer et al. 2010). In case of cadmium hyperaccumulation, however, the problem seems to be less studied. Hitherto, only four species are known to hyperaccumulate cadmium. Since these plants are also zinc hyperaccumulators, it suggests a common genetic basis of both phenomena (Verbruggen et al. 2009). Nonetheless, little is known regarding correlation between hyperaccumulation of zinc and cadmium.

Zinc and cadmium belong to the most intensively studied metals in terms of their impact on plants. Zinc is a micronutrient essential to plant growth; however, its excess can cause toxic effects. Cadmium is one the most frequent and the most dangerous inorganic pollutants. Although both metals share similar chemical properties (they have similar atomic radius, similar oxidation state in chemical compounds, and share similar geochemical properties), cadmium shows higher tendency to bond with sulfur and higher mobility in soils and in whole ecosystems (Emsley 1991; Kabata-Pendias 2010). It has been hypothesized that both metals can share similar pathway while entering the plant organism (Zhao et al. 2006). In contrast to zinc, which is essential for plant growth, cadmium is not found in any natural chemical compound in living organisms. Cadmium is widely studied in the context of environmental pollution and its’ impact on human health (di Toppi and Gabbrielli 1999). Although the metal is toxic to plants, it is known for its easy uptake (Kabata-Pendias 2010). Strong affinity to sulfhydryl groups is one of the most important biochemical characteristics of cadmium. The metal, however, can also easily bind to functional groups containing nitrogen or oxygen (Polatajko et al. 2007).

Research on metal-chelating compounds in organisms responsible for the metal homeostasis of the cell showed that different chemical compounds can be involved in this process including amino acids (e.g., histidine, nicotiamine), organic acids such as: malate and citric acid, phytochelatins, and metallothioneins (Szpunar 2005; Fenik et al. 2007; Polatajko et al. 2007; Maestri et al. 2010). Braude et al. (1980) mentioned that cadmium shows a tendency to accumulate in a protein fraction within the plant cell. This is particularly interesting, as most of the proteins regulating metal homeostasis of the plant cell, are also involved in detoxification, regulation of the cell cycle, proliferation, and apoptosis (Garcia et al. 2006). To understand thoroughly plant response to environmental stress caused by metal ions, it is necessary to localize, identify, and quantify metal-containing macromolecules. This task is particularly challenging from the analytical point of view. Hyphenated techniques for biological systems such as size exclusion chromatography coupled with plasma-mass spectrometry (SEC-ICP-MS) or laser ablation inductively coupled plasma-mass spectrometry (LA-ICP-MS) can be regarded as a solution of this problem. Recently, this technique was used for direct ablation of polyacrylamide gels (PAGE) (Szpunar 2005; Fenik et al. 2007; Polatajko et al. 2007).

We employed these techniques in order to investigate the influence of the Cd stress on mineral nutrient uptake and metal-binding proteins content in *A. halleri*.

We tested the following hypotheses:

1. *A. halleri* is a Cd hyperaccumulator.
2. There is a correlation between Cd uptake and the content of mineral nutrients.
3. Cd ions are bound by low-molecular-weight (<100 kDa) metal-binding proteins.

## Materials and Methods

### Plant Material

Experiments were carried out using *A*. *halleri* plants. Seeds were obtained from the Pb/Zn mining area in Boleslaw near Olkusz (S Poland), a region highly polluted with heavy metals (Przedpelska and Wierzbicka 2007; Abratowska et al. 2012). Plants were cultivated for 40 days in growth chamber under controlled temperature conditions (24 ± 4 °C), relative humidity of 65 ± 4 %, light intensity of ∼120 μmol/m^2^/s, and photoperiod of 8:16 h.

### Plant Cultivation and Metal Exposure

Seeds collected from the field were germinated in Petri dishes on filter paper moistened with diluted Knop nutrient solution (1:8 dilution) supplemented with A-Z mixture of trace elements (Strebeyko 1967), in the light at the temperature of 24 °C ± 1 °C. About 7 days old, seedlings were transferred into pot boxes containing perlite as a substrate. The perlite was washed daily with Knop’s nutrient solution of the following chemical composition—200 g/l Ca(NO_3_)_2_ × 4H_2_O, 71.5 g/l KNO_3_, 35.5 g/l KCl, 71.5 g/l MgSO_4_, 71.5 g/l KH_2_PO_4_, 28 g/l EDTA-Fe, and trace elements (including Zn^2+^ at the concentration of 0.4 μM), pH = 6.

The plants were divided into control group and two experimental groups (30 plants each). Experimental groups were treated every second day with cadmium added to the nutrient solution as Cd(NO_3_)_2_ × 4 H_2_O to achieve the concentrations of 45 and 225 μM Cd^2+^. Cd concentrations employed were chosen on the basis of pilot experiments (data not shown). We intended to use Cd doses that would enable plants to accumulate maximal amount of Cd in their tissues without visible effects of toxicity. Forty-day-old plants were harvested, divided into shoot and root portions, and then washed several times in distilled water. By the shoot portions, all the aerial portions of plant including stem and leaves are meant. To ensure complete removal of all the components of the growth medium (including Cd^2+^), roots were washed for 5 min in 20 mM EDTA. Subsequently, samples were immediately frozen in liquid nitrogen and ground using a pestle and mortar. To avoid protein unfolding and a loss of metals from their native structures, samples were placed on dry ice after grounding. A part of the sampled material was used in protein extraction, and the remaining portion was lyophilized.

### Quantification of Cd, Zn, and Mineral Nutrients

For quantification of the total metal content, 0.2 g of lyophilized leaf and 0.1 g of root samples were digested with 6 mL of a HNO_3_–H_2_O mixture (5:1) using a microwave-assisted digestion with closed vessels (Anton Paar, Graz, Austria). Triplicate extracts from all the samples were taken. Digested samples were diluted with Milli-Q water to 50 mL and analyzed by ICP-MS using an external calibration curve with Rh (10 ng mL^−1^) as an internal standard. The double focusing sector field ICP-MS (ELEMENT 2, ThermoFisher Scientific, Bremen, Germany) coupled to the Cetac autosampler (ASX-500, Omaha, Nebraska, USA) was used for multi-element analysis (Cd, Zn, Mg, S, Mn, Fe, Cu, Mo). Translocation index was calculated as described by Branquinho et al. (2007). Statistical significance of the observed differences between the control and experimental groups was tested by Kruskal–Wallis test using Statistica 9.1. Shape of the distribution within analyzed groups and the variance were checked prior to the analysis in order to ensure that the assumption of the homoscedasticity of the data is not broken. To test for correlations between cadmium and other analyzed elements, we used coefficient of determination (*R* ^2^). Regression lines and 95 % confidence intervals were calculated. Significance tests were carried out at 5 % significance level. All the statistical analyses were performed using Statistica 9.1 (Statsoft Inc.)

### Protein Extraction

Proteins were extracted from shoots and roots of the control and Cd-treated plants following the sample preparation protocol—5 g of leaves and 3 g of roots were homogenized with 6 mL of 50 mM HEPES-NaOH (pH 7.6) containing 2 % PVP and protease inhibitor cocktail (Complete, EDTA-free) using a ultrasonic probe homogenization technique (Branson, SONIFIER, Schwäbisch Gmünd, Germany). The homogenization method involved 5 × 30-s treatments until a well-homogenized extract was obtained. In order to obtain cytosol free from organelle samples were centrifuged at 105,000*g* for 1 h at 4 °C using a Sorvall Discovery 90SE ultracentrifuge (ThermoFisher, Dreieich, Germany). The supernatant was then centrifuged for 30 min at 4,000 rpm using a Heraeus SEPATECH centrifuge (ThermoFisher, Dreieich, Germany). The aliquots were stored at −80 °C for further investigations. The method employed has been optimized by Polatajko et al. (2011).

### SEC-ICP-MS Analysis of Leaves and Roots Extracts

Cadmium–protein complexes from shoots and roots extracts were probed by size exclusion chromatography (SEC, Dionex, Germering, Germany) coupled to ICP-MS (ELEMENT 2, Thermo Fisher Scientific, Bremen, Germany). SEC-purified fraction was also used for identification of Zn- and Cu-proteins. The method employed has been optimized by Polatajko et al. (2011).

### PAGE-LA-ICP-MS Analysis of Leaves and Roots Extracts

The separation of cadmium-containing proteins was done by using anodal native 10 % polyacrylamide gel electrophoresis (1D AN-PAGE) with a discontinuous Tris/Tricine system according to the manufacturer’s instructions. A Nd:YAG laser (Minilight II, Continuum, Santa Clara, USA) coupled to the sector filed ICP-MS was used for laser ablation (LA) of the Cd-binding proteins on the membrane. For a detailed description of the background of the chemical analyses, the reader is referred to Polatajko et al. (2007).

## Results

### The Influence of Cadmium on Biomass Production

Cd-treated plants showed no signs that could be associated with the toxic effect of Cd ions. Leaves showed no signs of chlorosis or necrosis; tissues were well hydrated (no signs of decreased turgidity). No signs of growth inhibition or differences in development were recorded between control and treated plants. Mean dry weight of shoots was 2.2, 1.8, and 1.65 g for the control and two experimental groups, respectively. Mean dry weight of roots was 0.3, 0.3, and 0.27 g, respectively. Nevertheless, there was no significant difference in biomass production between control and treated plants, regardless of the concentration of Cd in the growth medium (Kruskal–Wallis test, *p* = 0.1955 for shoot samples, *p* = 0.3594 for root samples).

The above-mentioned observations show that *A. halleri* is tolerant to tested cadmium doses. The fact that no signs of toxicity were observed while *A. halleri* accumulated Cd in its tissues in high amounts (particularly in shoots, see below) additionally confirms this notion.

### Cd and Zn Accumulation

**Fig. 1:**
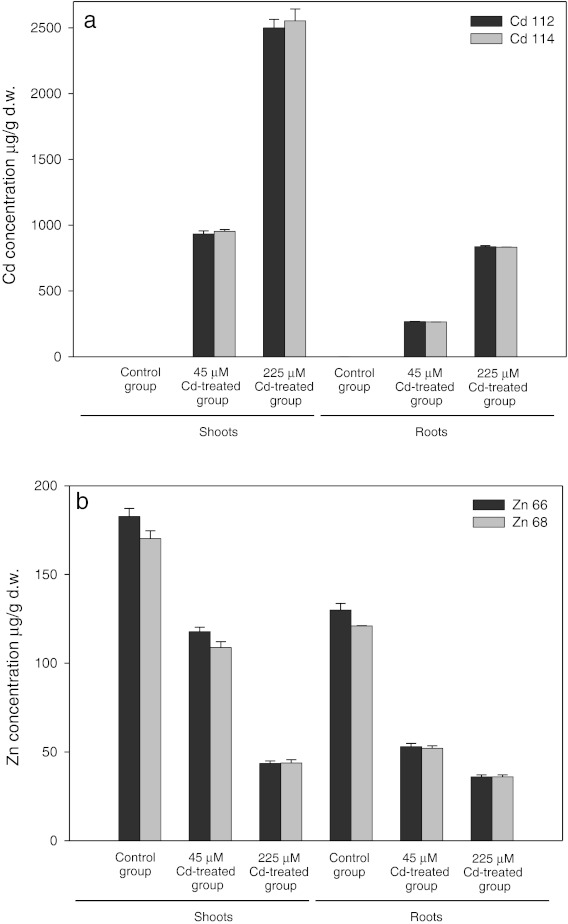
Total concentration of Cd (**a**) and Zn (**b**) in *A*. *halleri* extracts from shoots and roots, respectively. Metal concentration was quantified using ICP-MS

Results obtained for quantitative multi-element analysis relative to the dry mass are presented in Fig. 1a, b. Results showed that cadmium concentration in experimental plants (40 days of treatment) increased with increasing concentration of the metal in growth medium in leaves as well as in roots (Fig. 1a). In plants treated with lower dose of cadmium (45 μM Cd^2+^), concentration of the metal in roots was 265 μg g^−1^d.w., whereas in plants treated with the higher dose (225 μM Cd^2+^), it was 835 μg g^−1^d.w. We found that in treated plants cadmium concentration in shoots was 940 μg g^−1^d.w. and 2,525 μg g^−1^d.w, respectively, for lower and higher dose. Total concentration of the metal in treated plants was always higher in shoots than in roots.

The translocation index, calculated by dividing the concentration of the metal in shoots by the concentration of the metal in roots, varied narrowly between 3 and 3.5 for both tested cadmium doses.

Experiments showed that that zinc content in cadmium-treated plants was significantly lower (Fig. 1b). While in the control group zinc content was 175 μg g^−1^d.w., in treated plants, it decreased by 37 % and 70 % in case of lower (45 μM Cd^2+^) and higher (225 μM Cd^2+^) cadmium dose, respectively. In roots, zinc content followed the same pattern being the highest in control plants (125 μg g^−1^d.w.) and decreasing by 58 % and 71 % in both cadmium-treated groups. The Zn root–shoot translocation index calculated for each experimental group was 1.2 and 2.1, respectively.

The values of the translocation indices showed hyperaccumulation of both tested metals (Cd and Zn) by *A. halleri.* There was no significant difference in accumulation between investigated isotopes: ^112^Cd vs. ^114^Cd and ^66^Zn vs. ^68^Zn.

### The Relationships Between Cd and Mineral Nutrient Concentrations in Shoots and Roots

Statistical analysis showed the presence of significant correlations between the concentration of cadmium and other macro- (Mg, S) and microelements (Mn, Fe, Cu, Zn, Mo). Correlation analysis uncovered that significant and strong negative correlation exists between concentrations of zinc and cadmium (*R* ^2^ = −0.8487, *p* < 0.05 in roots; *R* ^2^ = −0.9922, *p* < 0.05 in shoots). Magnesium showed significant correlation with cadmium concentration in both roots and shoots, however, in roots, the coefficient of determination showed negative (*R* ^2^ = −0.9125, *p* < 0.05), whereas in shoots positive (*R* ^2^ = 0.8765, *p* < 0.05) value. No other investigated element showed significant correlation with cadmium concentration in roots at the significance level of 5 %. In shoots, significant correlations were also found between cadmium and manganese (*R* ^2^ = 0.9593, *p* < 0.05), cadmium and iron (*R* ^2^ = −0.8046, *p* < 0.05), as well as between cadmium and copper (*R* ^2^ = −0.7307, *p* < 0.05). There was no significant correlation between cadmium and sulfur (*R* ^2^ = 0.2593, *p* > 0.05 in roots, *R* ^2^ = 0.1064, *p* > 0.05 in shoots). Regression lines along with coefficients of determination (*R* ^2^) and results of significance testing for each analyzed element can be found in electronic supplementary material (Fig. ESM 1, Fig. ESM 2).

### Cd-Binding Proteins

Using ICP-MS, we measured the content of Cd-binding proteins in shoots and roots. We found that, in plants treated with 45 μM Cd, the content of Cd-binding proteins was about 8.6 and 9.6 μg/g f.w. in shoots and roots, respectively. In plants treated with 225 μM Cd, these values were about 52.5 and 33.5 μg/g f.w., respectively. We did not record the presence of Cd-binding proteins in the control group.

**Fig. 2:**
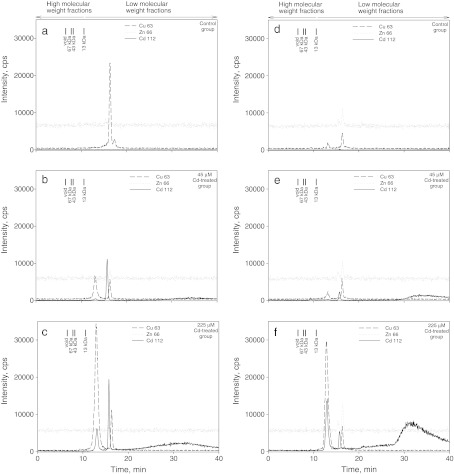
**a-f** SEC-ICP-MS electropherogram of ^112^Cd^+^, ^66^Zn^+^, and ^64^Cu^+^ intensity in *A*. *halleri* extracts from roots [control plants (**a**), plants treated with 45 μM Cd (**b**), plants treated with 225 μM Cd (**c**)], and shoots [control plants (**d**), plants treated with 45 μM Cd (**e**), plants treated with 225 μM Cd (**f**)]. The signal from ^112^Cd^+^ is designated by the *solid line*; the signal from ^66^Zn^+^ is designated by the *dashed line*, and the signal from ^64^Cu^+^ is designated by the *dotted line*

The control electropherograms (Fig. 2a, d) showed the lack of Cd-binding proteins in both shoots and roots. Application of SEC-ICP-MS enabled us to detect one signal for Cd-binding protein in roots (Fig. 2b) as well as in shoots (Fig. 2e), during the examination of the plant material treated with 45 μM Cd^2+^. The same methodology enabled us to detect two signals and one broad peak showing the presence of Cd-binding proteins in roots and shoots obtained from plants treated with 225 μM solution of cadmium (Fig. 2c, f). All the identified Cd-proteins had small molecular weight of <13 kDa. Apart from Cd-proteins, two clear signals for Zn-binding proteins were also recorded in shoot extracts from of Cd-treated plants (Fig. 2a–f). We also recorded two signals of Cu-binding proteins in all the analyzed extracts (Fig. 2a–f). One of the Cu-binding proteins was eluted together with one of the Cd-binding protein (Fig. 2c, f). Both Cu-binding proteins identified by us showed low molecular weight of <13 kDa. It should be noted that intensity of the signal from the bigger protein increased with the increase of cadmium content in growth medium in samples from roots and shoots. The intensity of the signal from the smaller Cu-binding protein was smaller in Cd-treated than in control plants in roots. In shoots, signal intensity from the smaller molecule did not differ so strongly. We noticed that intensity of the signal from Zn-binding protein increased slightly in samples treated with the highest dose of cadmium.

**Fig. 3:**
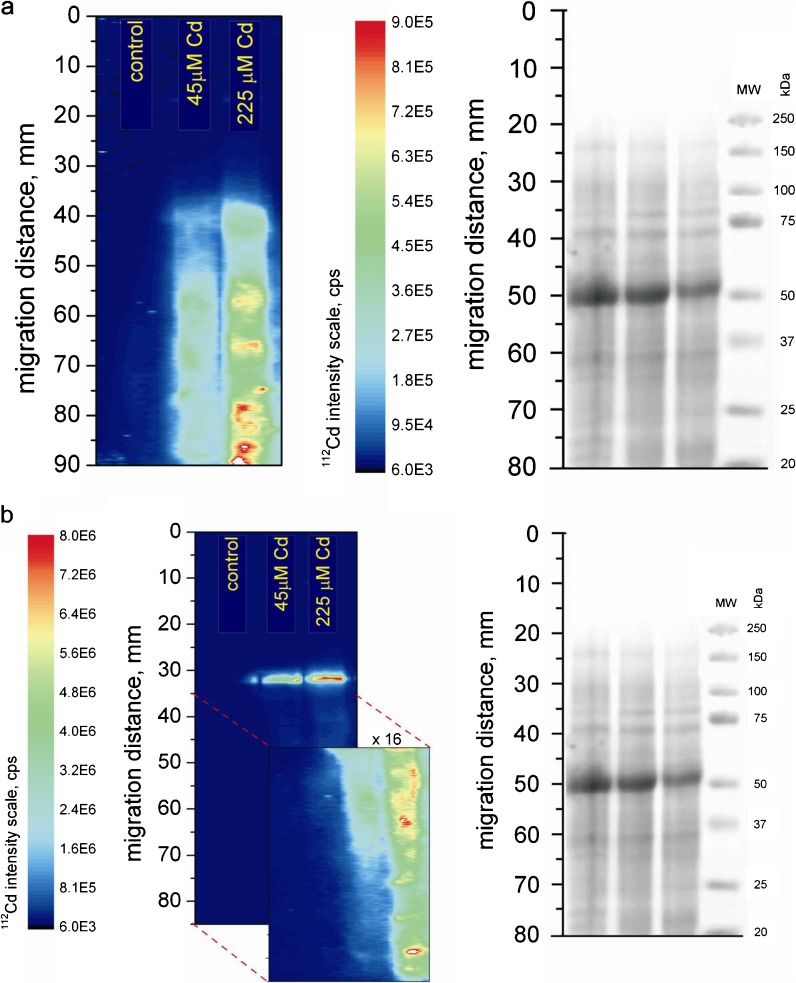
SDS-PAGE gel stained with Coomassie blue (*right panels*) and ^112^Cd^+^ intensity for PAGE-LA-ICP-MS (*left panels*) of *A*. *halleri* extracts from roots (**a**) and from shoots (**b**)

Application of PAGE-LA-ICP-MS (laser ablation inductively coupled plasma-mass spectrometry used for ablation of polyacylamide gels) enabled us to record Cd-binding proteins with higher resolution than the one offered by SEC-ICP-MS (Fig. 3a, b). Figure 3a presents electropherogram of Cd-binding proteins in roots. In samples treated with 45 μM Cd^2+^, signal below 75 kDa is smeared and looks like a distribution of the metal through the gel. In roots of plants treated with 225 μM Cd^2+^, four bands at 35, 25, 20, and ∼19 kDa were recorded. In leaves from Cd-treated plants (regardless the dose applied), one band at ∼75 kDa was recorded (Fig. 3b). Additionally, two bands at 50 and 20 kDa were recorded in leaf extracts from plants treated with 225 μM Cd^2+^ (Fig. 3b).

We showed for the first time that Cd-binding proteins with low molecular weight (<100 kDa) are present in *A. halleri* and that the amount of Cd bound to these proteins correlated with the amount of the metal in the growth medium. The presence of two Zn- and two Cu-binding proteins were recorded in control and metal-exposed plants.

## Discussion

### Experimental Conditions

Our experiments were carried out employing perlite and relatively long metal treatment using cadmium doses mimicking concentrations of this metal found in polluted soils (Nriagu and Pacyna 1988; Nriagu 1996; Yanai et al. 2006). During pilot experiments (data not shown), we tried to adjust cadmium dose in such a way as to avoid the emergence of visible toxic effects like chlorosis, necrosis, or decreased biomass production. Di Toppi and Gabbrielli (1999) indicate that growth conditions play crucial role in experiments focused on cadmium tolerance in plants. According to them, Cd tolerance in higher plants is a natural or artificially given capacity, regulated by interactions between genetic and environmental factors, to bear high levels of Cd exposure for a long time, without appreciable detrimental effects on metabolism. It seems that employing short-term Cd treatment leads to activation of more or less efficient detoxification mechanism that is present in all higher plants (di Toppi and Gabbrielli 1999). Application of long-term Cd treatment (i.e., chronic stress) gives an opportunity to get insights into plant response in which all the homeostatic mechanisms of the cell are involved. In that way, we are able to assess the real level of Cd tolerance in a given species. From all the above-mentioned reasons, we decided to employ long-term (40 days) treatment with cadmium spanning almost the entire life cycle of investigated plants (from a seedling to the mature organism).

### Cd Accumulation

Although *A. halleri* is a well-known zinc hyperaccumulator, its status as a cadmium hyperaccumulator was uncertain until recently. It has been shown that *A. halleri* has a potential to hyperaccumulate cadmium (Dahmani-Muller et al. 2000; Bert et al. 2002). Recently, a growing number of experimental studies confirms the ability of the plant to hyperaccumulate cadmium (Bert et al. 2003; Cosio et al. 2004; Zhao et al. 2006; Verbruggen et al. 2009; Maestri et al. 2010). The majority of above-mentioned studies, however, were carried out in hydroponics and employed short-term cadmium exposure in high doses.

Our results have shown that *A. halleri* is able to accumulate cadmium up to 1,000 μg g^−1^d.w. in roots and up to 2,500 μg g^−1^d.w. in shoots without any visible symptoms of phytotoxicity. Similar Cd content in shoots (2,700 mg kg^−1^d.w.) was obtained by Küpper et al. (2000) under laboratory conditions. Interestingly, however, Küpper et al. (2000) and Bert et al. (2003) showed that Cd content in shoots was lower than in roots. Similar pattern of Cd translocation has been observed in *Thlaspi caerulescens* by Lombi et al. (2000) and Zhao et al. (2006). We suppose that difference in the pattern of Cd translocation might have been caused by different growing conditions. Küpper et al. (2000), Lombi et al. (2000), Bert et al. (2003), and Zhao et al. (2006) carried out their experiments employing hydroponic approach, while we have grown plants using perlite as a semi-hydroponic culture system and long-term cadmium exposure.

Moreno-Jimenez et al. (2007) have summarized results concerning different patterns of mercury translocation in plants depending on the type of substrate used during cultivation. They showed that root to shoot translocation of the metal was significantly higher in plants grown in perlite when compared with those grown under hydroponic conditions. Our data seem to support these observations.

Also, Vazquez and Carpena-Ruiz (2005), Moreno-Jimenez et al. (2007), and Sobrino-Plata et al. (2009) have noticed that the type of substrate used during cultivation significantly affects the uptake of heavy metals. The abovementioned authors paid particular attention to the differences between classic hydroponics and perlite-based cultivation system. It seems that growing plants in almost completely inert substrate such as perlite mimics well the conditions for root growth found in natural soils (Olympios 1999; Robbins and Evans 2005) including adequate humidity, stimulation of root growth, etc. (Moreno-Jimenez et al. 2007; Sobrino-Plata et al. 2009). At the same time, access to nutrients and heavy metals is more limited under perlite cultivation than in pure hydroponic conditions (Sobrino-Plata et al. 2009). Küpper et al. (2000), Lombi et al. (2000), Bert et al. (2003), and Zhao et al. (2006) showed that *A. halleri* accumulated Cd in the amounts higher than 0.01 % d.w., what has been suggested as a criterion for Cd hyperaccumulation by Brooks (1998). Our results agree with these findings. We have also shown, however, that the plant is able to translocate the metal from roots to shoots in such a way that the ratio of the metal content in roots and in shoots is higher than 1. This criterion of metal hyperaccumulation has been proposed by McGrath and Zhao (2003).

### The Correlation Between Cd and Zn Hyperaccumulation

Cd hyperaccumulation has been shown in all known Zn hyperaccumulating plants. This suggests that genetic basis of both phenomena might be, at least partially, the same (Verbruggen et al. 2009). Our results indicate negative correlation between Cd and Zn content in both shoots and roots in *A. halleri*. The higher content of cadmium was recorded; the lower was the content of zinc.

The same phenomenon, suggesting a competition between zinc and cadmium, has been also observed by Zhao et al. (2006). On the basis of an experiment carried out during 3 weeks under hydroponic conditions, they demonstrated that increase in Zn content in the growth medium caused decrease in Cd content in plants. Investigating the effect of Zn and Cd treatment on the content of these metals in plant tissues, Küpper et al. (2000) showed that increase in Zn concentration caused the decrease of cadmium content in shoots by 50 %. The existence of a competition between Zn and Cd has been also evidenced by Cosio et al. (2004), who demonstrated that increased amount of Cd in the growth medium reduced Zn accumulation.

An important difference between the authors cited above and our experiments is that, in our study, concentration of Zn in the growth medium remained on constant level of 0.4 μM and was similar to the concentration found in natural, unpolluted soils (Grimme 1968).

Our results seem to support the hypothesis put forward by Zhao et al. (2006) that *A. halleri* is able to hyperaccumulate Cd through the Zn pathway and that, in case of increased Cd content in the growth medium, Zn is accumulated in lower amounts due to the competition between both metals.

### The Correlation Between Cd and Mineral Nutrient Accumulation

It has been shown that the influence of cadmium on the content of mineral nutrients in plants can depend on the concentration of cadmium, growth conditions, species, or ecotype being investigated or even on plant organ under study (Zhang et al. 2002; Cai et al. 2010). Interactions between cadmium and essential mineral elements have been studied in potatoes (Gonçalves et al. 2009), different ecotypes of wheat (Zhang et al. 2002), several Cd-tolerant, and non-tolerant ecotypes of rice (Liu et al. 2003; Cai et al. 2010), in *Betula pendula*—a pioneer species colonizing post-industrial polluted areas (Gussarsson et al. 1996) as well as in cadmium hyperaccumulators: *Brasica juncea* (Jiang et al. 2004), *T*. *caerulescens* (Roosens et al. 2003), and *Sedum alfredii* (Yang et al. 2004). This wide literature reveals, however, a very complicated and often contradictory picture of the problem.

In the present work, we show for the first time that cadmium influences the uptake of mineral nutrients in *A*. *halleri*. Our data show that the content of cadmium and mineral nutrients (Mg^2+^, Mn^2+^, Fe^2+^, Cu^2+^, and Zn^2+^) was significantly correlated. At the same time, the content of mineral nutrients recorded by us in investigated plants ranged within normal limits for wild plants (Kabata-Pendias 2010). It seems from the above-mentioned reasons that correlations between the content of cadmium and mineral nutrients might not be an effect of a simple specific competition between these elements but rather an outcome of the mechanism involved in maintaining ion homeostasis. It can be hypothesized that the ability to tolerate and hyperaccumulate heavy metals by *A. halleri* may result from the ability of the species to maintain ion homeostasis under cadmium stress.

### Analysis of Metal-Binding Proteins

It is estimated that about 40 % of all proteins contain heavy metal ion in their structure (Garcia et al. 2006). Metal-containing proteins may be divided into two groups: metalloproteins and metal-binding proteins. The first group is characterized by strong affinity between protein and metal ion. The second group shows significantly weaker affinity between the two types of molecules. This fact makes the analysis of metal binding proteins an extremely difficult task as metals can be easily lost during the procedure of sample preparation. Hence, it is necessary here to employ chemical techniques of high resolution for protein separation and identification. In our research, we used SEC-ICP-MS and PAGE-LA-ICP-MS. Both methods are widely accepted as useful techniques for bioinorganic speciation analysis (Szpunar 2005; Garcia et al. 2006; Polatajko et al. 2007, 2011). SEC-ICP-MS enabled us to identify dominant coordination complexes of cadmium of molecular weight lower than 13 kDa in treated plants. New-generation columns applied in SEC-ICP-MS enable the researcher to identify intact coordination complexes (Persson et al. 2006). The multi-elemental capacity of the method is one of its major advantages (Persson et al. 2006; Polatajko et al. 2011). We employed this tool to elucidate the problem whether, apart from Cd, other important elements (such as Zn and Cu) can also interact with proteins. In our case, the use of SEC-ICP-MS enabled us to identify proteins forming coordination complexes with the following isotopes: ^112^Cd, ^66^Zn, ^63^Cu. SEC-ICP-MS has, however, some limitations in resolution when compared with other techniques (Persson et al. 2006). The application of size exclusion chromatography together with inductively coupled plasma mass spectrometry enabled us, however, to detect and identify metal binding proteins in *A. halleri*.

The application of PAGE-LA-ICP-MS enabled us to identify more cadmium-binding proteins with higher molecular weight, alongside with relatively small cadmium loss during experimental procedures. Cd-binding proteins identified using this method ranged between 19 and 75 kDa. It is now widely accepted that PAGE-LA-ICP-MS is a powerful tool in research on metal binding complexes in plant material rich in proteins (Polatajko et al. 2011). One important limitation of the technique is, however, that multi-elemental analysis is not possible on a single-blot membrane. The method, however, allowed the detection of some rare Cd-proteins with resolution significantly better than the one offered by SEC-ICP-MS. These investigations allowed us to show that long-term cadmium exposure alters the protein composition of a plant.

Investigation of metal-binding proteins requires a lot of effort and the use of advanced methods. Studies in this subject should be focused on the structure and biological role of these proteins. It seems to us that the first and the most important step on this way is to localize and quantify the content of metal-binding macromolecules and to conduct their preliminary identification. Our research is an important contribution to the problem.

### Metal-Binding Proteins

We showed for the first time that control and Cd-treated plants of *A. halleri* differ significantly at molecular level. Studies carried out hitherto were focused mainly on the isolation and identification of Cd-binding proteins (e.g., Bartolf et al. 1980; Rauser 1984; Kumar and Prasad 2004; Kastenholz 2006; Fenik et al. 2007; Polatajko et al. 2007, 2011). Our study enabled us not only to identify these proteins but also to show that the pattern of their occurrence differ between control and Cd-treated plants, as well as between groups treated with different doses of cadmium. These differences were shown in plants without any signs of Cd toxicity. We think that this finding gives a new perspective on the phenomenon of metal hyperaccumulation.

In Cd-treated plants, we identified Cd-binding proteins of low molecular weight (LMW): several proteins of <13 kDa, proteins of 20, 25, and 50 kDa, and of about 19, 35, 45 and 75 kDa in size. In control plants, we did not record the presence of these molecules, which can suggest that Cd-binding proteins are involved in plant response to cadmium. Fenik et al. (2007) showed the presence of numerous Cd-binding proteins in Cd-resistant lines of *Nicotiana plumbaginifolia*. They showed the presence of LMW Cd-binding proteins of 19, 34, and 40 kDa and five proteins of <13 kDa in size in Cd-treated plants. In cadmium-sensitive *A*. *thaliana*, the presence of high-molecular-weight Cd-binding protein of approximately 200 kDa in size has been recorded by Kastenholz (2006). The presence of LMW Cd-binding proteins in Cd-tolerant ecotypes treated with cadmium can be considered as an indirect proof of the significance of these molecules in plant response to cadmium stress.

Our experiments showed that the amount of Cd-proteins correlated with the amount of Cd present in the growth medium. Study carried out by Kumar and Prasad (2004) showed also that there is a positive correlation between Cd bioaccumulation and the concentration of Cd-binding proteins. Hence, application of long-term Cd exposure instead of short-term, “acute” treatment seems to be more appropriate to the authors, as it should promote Cd-protein synthesis.

Our results evidenced also the presence of LMW (<13 kDa) Cu- and Zn-binding proteins in *A. halleri*. It suggests the presence of specific proteins with very low molecular weight and high affinity to divalent metal ions in investigated plants. It seems that two metabolically important metal ions (Cu^2+^ and Zn^2+^) can form complexes with proteins similar to Cd-binding proteins, as they were characterized by almost identical elution time in size exclusion chromatography. On the basis of our studies, however, it is difficult to answer whether Cd, Cu, and Zn bind to the same or different proteins.

Identification of Cd-binding proteins in *A. halleri* can be of importance in our understanding of the mechanisms of cadmium tolerance and hyperaccumulation in plants. It has been shown that Cd-binding proteins can contain cysteine or histidine (Mejare and Bulow 2001). Both amino acids form complexes with metal ions relatively easily by binding to the thiol group in case of the former or to nitrogen from imidazole functional group (that can easily form coordination bonds with metal cations) in case of the latter.

It is known that cysteine-rich proteins such as metallothioneins and phytochelatins form stable complexes with cadmium (Mejare and Bulow 2001; Fenik et al. 2007; Meyer et al. 2011). Hitherto, numerous studies stressed the importance of phytochelatins (oligomers of glutathione) in plant response to heavy metals (e.g., di Toppi and Gabbrielli 1999; Szpunar 2005; Memon and Schroder 2009; Meyer et al. 2011). It is usually accepted that molecular weight of Cd–phytochelatin complexes range between 2.5 and 10 kDa, depending on the plant species and physiological status of investigated specimens (Persson et al. 2006). It is also widely accepted that this mechanism of Cd detoxification plays a key role in Cd-sensitive ecotypes (Ebbs et al. 2002). In case of tolerant plants, or Cd hyperaccumulators such as *A. halleri*, the role of phytochelatins is almost negligible (Ebbs et al. 2002; Fenik et al. 2007; Meyer et al. 2011). It seems, therefore, that amino acids in the form of glutathione could be involved in the protection against oxidative stress caused by heavy metal ions. In this case, other molecules have to act as detoxification agents. For all the above-mentioned reasons, it seems to us that Cd-binding proteins of molecular weight <13 kDa cannot be interpreted as phytochelatins.

Metallothioneins are another group of chemical compounds that are thought to play role in plant resistance to cadmium (e.g., Mejare and Bulow 2001). Molecular weight of Cd–metallothionein complexes range between 6 and 7 kDa (Sabolic et al. 2002). Mejare and Bulow (2001), however, put forward the hypothesis that the expression of cysteine-free metal chelating proteins is less toxic for cells than the expression of cysteine-rich molecules. According to them, this scenario is particularly relevant for plants in which synthesis of such molecules is controlled by a constitutive promoter and it is not adjusted by the metal concentration inside the cell (Mejare and Bulow 2001). Moreover, Gussarsson (1994) showed that increase in sulfur content under cadmium stress was correlated with the increased content of some Cd-binding peptides containing sulfur. We showed that sulfur content in treated plants was not correlated with Cd. Therefore we think, that molecules of the molecular weight < 13 kDa cannot be associated with metallothioneins. Hence, there are premises that, in small proteins identified by us, Cd is not bound by sulfhydryl groups that are common in phytochelatins and metallothioneins.

It is widely known that histidine can act as a very efficient ligand for metal ions such as Cd^2+^, Zn^2+^, Cu^2+^, Ni^2+^, and Co^2+^(Wierzbicka et al. 2007; Verbruggen et al. 2009). This amino acid is also thought to be one of the most important compounds responsible for the chelation of metal ions in hyperaccumulators (Haydon and Cobbett 2007; Verbruggen et al. 2009). Additionally, our previous research on the speciation of Cd in *Allium cepa* epidermal cells showed the presence of specific cadmium ligand—the histidine (Wierzbicka et al. 2007). This can suggest that LMW proteins identified by us and able to bind Cd could contain histidine—the amino acid playing a key role in heavy metal detoxification in hyperaccumulators. This hypothesis requires, however, further studies and evaluation.

It is worth noting that all the investigated plants showed high level of Cd-tolerance and differed significantly in terms of the pattern of Cd-binding proteins. This can suggest that these proteins can be involved in cadmium detoxification as it was suggested previously by several authors (Fenik et al. 1995; Woolhouse 1983; Meyer et al. 2011).

## Conclusions

We showed that *A. halleri* is able to translocate Cd to aerial parts in high amounts (translocation index >1) that confirmed its status as Cd hyperaccumulator. Our experiments showed that Cd influenced the uptake of different mineral nutrients. Our results suggest that Cd and Zn can be hyperaccumulated by *A. halleri* through a common pathway. The data obtained by us point out that Cd is bound by low-molecular-weight metal-binding proteins of the molecular mass <100 kDa. These proteins are unlikely to be phytochelatins or metallothioneins. We hypothesize that these proteins can play a role in broadly understood Cd detoxification process in *A. halleri*.

## Electronic supplementary material

Below is the link to the electronic supplementary material.

Fig. ESM1 a-h Scatter plots showing correlations between Cd and mineral nutrient content in *A. halleri* roots. Regression lines (*solid line*), 95 % confidence intervals (*dotted lines*), coefficient of determination (*R* ^2^), and the equation of the regression line were given for each element. **a** ^114^Cd vs^. 112^Cd, **b** ^24^ Mg vs^. 112^Cd, **c** ^32^ S vs^. 112^Cd, **d** ^55^Mn vs^. 112^Cd, **e** ^58^Fe vs^. 112^Cd, **f** ^63^Cu vs^. 112^Cd, **g** ^66^Zn vs^. 112^Cd, **h** ^98^Mo vs^. 112^Cd. Element concentrations were quantified using ICP-MS (PDF 93 kb)

Fig. ESM2 a-h Scatter plots showing correlations between Cd and mineral nutrient content in *A. halleri* shoots. Regression lines (*solid line*), 95 % confidence intervals (*dotted lines*), coefficient of determination (*R* ^2^), and the equation of the regression line were given for each element. **a** ^114^Cd vs^. 112^Cd, **b** ^24^ Mg vs^. 112^Cd, **c** ^32^ S vs^. 112^Cd, **d** ^55^Mn vs^. 112^Cd, **e** ^58^Fe vs^. 112^Cd, **f** ^63^Cu vs^. 112^Cd, **g** ^66^Zn vs^. 112^Cd, **h** ^98^Mo vs^. 112^Cd. Element concentrations were quantified using ICP-MS (PDF 104 kb)

## Abbreviations

ICP-MS: Inductively coupled plasma-mass spectrometry
LA-ICP-MS: Laser ablation inductively coupled plasma-mass spectrometry
LMW: Low molecular weight
PAGE: Polyacrylamide gel electrophoresis
PVP: Polyvinylpyrrolidone
SEC-ICP-MS: Size exclusion chromatography coupled with plasma-mass spectrometry

## References

[CR1] Abratowska, A., Wąsowicz, P., Bednarek, P., Telka, J., & Wierzbicka, M. (2012). Morphological and genetic distinctiveness of the metallicolous and non-metallicolous populations of *Armeria maritima* s. l. (Plumbaginaceae) in Poland. *Plant Biology, 14*, 586–595. 10.1111/j.1438-8677.2011.00536.x 22243547

[CR2] Al-Shehbaz, I. A., & O’Kane, S. L., Jr. (2002). Taxonomy and phylogeny of *Arabidopsis* (Brassicaceae). *The Arabidopsis Book*. doi:10.1199/tab.0009. 10.1199/tab.0001 PMC3243115 22303187

[CR3] Bartolf M, Brenna E, Price CA (1980). Partial characterization of a cadmium-binding protein from the roots of cadmium-treated tomato. Plant Physiology.

[CR4] Bert V, Bonnin I, Saumitou-Laprade P, de Laguerie P, Petit D (2002). Do *Arabidopsis halleri* from nonmetallicolous populations accumulate zinc and cadmium more effectively than those from metallicolous populations?. New Phytologist.

[CR5] Bert V, Meerts P, Saumitou-Laprade P, Salis P, Gruber W, Verbruggen N (2003). Genetic basis of Cd tolerance and hyperaccumulation in *Arabidopsis halleri*. Plant and Soil.

[CR6] Branquinho C, Serrano HC, Pinto MJ, Martins-Loucao MA (2007). Revisiting the plant hyperaccumulation criteria to rare plants and earth abundant elements. Environmental Pollution.

[CR7] Braude GL, Nash AM, Wolf WJ, Carr RL, Chaney RL (1980). Cadmium and lead content of soybean products. Journal of Food Science.

[CR8] Broadley MR, White PJ, Hammond JP, Zelko I, Lux A (2007). Zinc in plants. New Phytologist.

[CR9] Brooks RR (1998). Plants that hyperaccumulate heavy metals: their role in phytoremediation, microbiology, archaeology, mineral exploration, and phytomining.

[CR10] Cai Y, Lin L, Cheng W, Zhang G, Wu F (2010). Genotypic dependent effect of exogenous glutathione on Cd-induced changes in cadmium and mineral uptake and accumulation in rice seedlings (*Oryza sativa*). Plant, Soil and Environment.

[CR11] Cosio C, Martinoia E, Keller C (2004). Hyperaccumulation of cadmium and zinc in *Thlaspi caerulescens* and *Arabidopsis halleri* at the leaf cellular level. Plant Physiology.

[CR12] Dahmani-Muller H, van Oort F, Gelie B, Balabane M (2000). Strategies of heavy metal uptake by three plant species growing near a metal smelter. Environmental Pollution.

[CR13] di Toppi LS, Gabbrielli R (1999). Response to cadmium in higher plants. Environmental and Experimental Botany.

[CR14] Ebbs S, Lau I, Ahner B, Kochian L (2002). Phytochelatin synthesis is not responsible for Cd tolerance in the Zn/Cd hyperaccumulator *Thlaspi caerulescenes* (J. and C. Presl). Planta.

[CR15] Emsley J (1991). Oxford chemistry guides the elements.

[CR16] Ernst W, Shaw J (1990). Mine vegetation in Europe. Heavy metal tolerance in plants: Evolutionary aspects.

[CR17] Fenik SI, Trofimyak TB, Blyum YB (1995). Mechanisms of development of plant tolerance to heavy metals. Uspekhisovremennoĭbiologii.

[CR18] Fenik SI, Solodushko VG, Kalinyak TB, Blume AB (2007). The role of Cd-binding proteins and phytochelatins in the formation of cadmium resistance in *Nicotiana plumbaginifolia* cell lines. Cytology and Genetics.

[CR19] Garcia JS, de Magalhaes CS, Arruda MAZ (2006). Trends in metal-binding and metalloprotein analysis. Talanta.

[CR20] Gode C, Decombeix I, Kostecka A, Wąsowicz P, Pauwles M, Courseaux A, Saumitou-Laprade P (2012). Nuclear microsatellite loci for *Arabidopsis halleri* (Brassicaceae), a model species to study plant adaptation to heavy metals. American Journal of Botany.

[CR21] Gonçalves JF, Antes FG, Maldaner J, Pereira LB, Tabaldi LA, Rauber R (2009). Cadmium and mineral nutrient accumulation in potato plantlets grown under cadmium stress in two different experimental culture conditions. Plant Physiology and Biochemistry.

[CR22] Grimme H (1968). Adsorption of Mn, Co, Cu and Zn by goethite from dilute solutions. Zeitschrift für Pflanzenernährung und Bodenkunde.

[CR23] Gussarsson M (1994). Cadmium-induced alterations in nutrient composition and growth of *Betula pendula* seedlings: The significance of fine root as a primary target for cadmium toxicity. Journal of Plant Nutrition.

[CR24] Gussarsson M, Asp H, Adalsteinsson S, Jensén P (1996). Enhancement of cadmium effects on growth and nutrient composition of birch (*Betula pendula*) by buthioninesulphoximine (BSO). Journal of Experimental Botany.

[CR25] Haydon MJ, Cobbett CS (2007). Transporters of ligands for essential metal ions in plants. New Phytologist.

[CR26] Jiang XJ, Luo YM, Liu Q, Liu SL, Zhao QG (2004). Effects of cadmium on nutrient uptake and translocation by Indian mustard. Environmental Geochemistry and Health.

[CR27] Kabata-Pendias A (2010). Trace elements in soils and plants.

[CR28] Kastenholz B (2006). Comparison of the electrochemical behavior of the high molecular weight cadmium proteins in *Arabidopsis thaliana* and in vegetable plants on using preparative native continuous polyacrylamide gel electrophoresis (PNC-PAGE). Electroanalysis.

[CR29] Kumar GP, Prasad MNV (2004). Cadmium-inducible proteins in *Ceratophyllum demersum* L. (a fresh water macrophyte): Toxicity bioassays and relevance to cadmium detoxification. Bulletin of Environmental Contamination and Toxicology.

[CR30] Küpper H, Lombi E, Zhao FJ, McGrath SP (2000). Cellular compartmentation of cadmium and zinc in relation to other elements in the hyperaccumulator *Arabidopsis halleri*. Planta.

[CR31] Lasat MM, Kochian LV, Flores HE, Lynch JP, Eissenstat D (1998). Physiological basis for Zn hyperaccumulation in *Thlaspicaerulescens*. Radical biology: Advances and perspectives on the function of plant roots.

[CR32] Lasat MM, Kochian LV, Terry N, Bañuelos G (2000). Physiology of Zn hyperaccumulation in *Thlaspi caerulescens*. Phytoremediation of contaminated soil and water.

[CR33] Liu JG, Liang JS, Li KQ, Zhang ZJ, Yu BY, Lu XL (2003). Correlations between cadmium and mineral nutrients in absorption and accumulation in various genotypes of rice under cadmium stress. Chemosphere.

[CR34] Lombi E, Zhao FJ, Dunham SJ, McGrath SP (2000). Cadmium accumulation in populations of *Thlaspi caerulescens* and *Thlaspi goesingense*. New Phytologist.

[CR35] Maestri E, Marmiroli M, Visioli G, Marmiroli N (2010). Metal tolerance and hyperaccumulation: Costs and trade-offs between traits and environment. Environmental and Experimental Botany.

[CR36] McGrath SP, Zhao FJ (2003). Phytoextraction of metals and metalloids from contaminated soils. Current Opinion in Biotechnology.

[CR37] Mejare M, Bulow L (2001). Metal-binding proteins and peptides in bioremediation and phytoremediation of heavy metals. Trends in Biotechnology.

[CR38] Memon AR, Schroder P (2009). Implications of metal accumulation mechanisms to phytoremediation. Environmental Science and Pollution Research.

[CR39] Meyer CL, Kostecka AA, Saumitou-Laprade P, Creach A, Castric V, Pauwels M, Frerot H (2010). Variability of zinc tolerance among and within populations of the pseudometallophyte species *Arabidopsis halleri* and possible role of directional selection. New Phytologist.

[CR40] Meyer CL, Peisker D, Courbot M, Craciun AR, Cazale AC, Desgain D (2011). Isolation and characterization of *Arabidopsis halleri* and *Thlaspi caerulescens* phytochelatin synthases. Planta.

[CR41] Moreno-Jimenez E, Penalosa JM, Esteban E, Carpena-Ruiz RO (2007). Mercury accumulation and resistance to mercury stress in *Rumex induratus* and *Marrubium vulgare* grown in perlite. Journal of Plant Nutrition and Soil Science.

[CR42] Nriagu JO (1996). A history of global metal pollution. Science.

[CR43] Nriagu JO, Pacyna JM (1988). Quantitative assessment of worldwide contamination of air, water and soils by trace-metals. Nature.

[CR44] Olympios CM (1999). Overview of soilless culture: Advantages, constraints and perspectives for its use in Mediterranean countries. Cahiers Options Méditerranéennes.

[CR45] Pauwels M, Frerot H, Bonnin I, Saumitou-Laprade P (2006). A broad-scale analysis of population differentiation for Zn tolerance in an emerging model species for tolerance study: *Arabidopsis halleri* (Brassicaceae). Journal of Evolutionary Biology.

[CR46] Persson D, Hansen TH, Holm PE, Schjørring JK, Cakmak I, Husted S (2006). Multi-elemental speciation analysis of barley genotypes differing in tolerance to Cd toxicity using LC-ICP-MS and ESI-TOF-MS. Journal of Analytical Atomic Spectrometry.

[CR47] Polatajko A, Azzolini M, Feldmann I, Stuezel T, Jakubowski N (2007). Laser ablation-ICP-MS assay development for detecting Cd- and Zn-binding proteins in Cd-exposed *Spinacia oleracea* L. Journal of Analytical Atomic Spectrometry.

[CR48] Polatajko A, Feldmann I, Hayen H, Jakubowski N (2011). Combined application of a laser ablation-ICP-MS assay for screening and ESI-FTICR-MS for identification of a Cd-binding protein in *Spinacia oleracea* L. after exposure to Cd. Metallomics.

[CR49] Przedpelska E, Wierzbicka M (2007). *Arabidopsis arenosa* (Brassicaceae) from a lead-zinc waste heap in southern Poland—A plant with high tolerance to heavy metals. Plant and Soil.

[CR50] Rauser WE (1984). Isolation and partial purification of cadmium-binding protein from roots of the grass *Agrostis gigantea*. Plant Physiology.

[CR51] Robbins JA, Evans MR (2005). Growing media for container production in a greenhouse or nursery: Part I (components and mixes).

[CR52] Roosens N, Verbruggen N, Meerts P, Ximénez-Embun P, Smith JAC (2003). Natural variation in cadmium tolerance and its relationship to metal hyperaccumulation for seven populations of *Thlaspi caerulescens* from western Europe. Plant, Cell & Environment.

[CR53] Sabolic I, Ljubojevic M, Herak KCM, Brow D (2002). Cd-MT cause endocytosis of brush-border transporters in rat proximal tubules. American Journal of Physiology. Renal Physiology.

[CR54] Sobrino-Plata J, Ortega-Villasante C, Flores-Caceres ML, Escobar C, Del Campo FF, Hernandez LE (2009). Differential alterations of antioxidant defenses as bioindicators of mercury and cadmium toxicity in alfalfa. Chemosphere.

[CR55] Strebeyko P (1967). Introduction to plant physiology.

[CR56] Szpunar J (2005). Advances in analytical methodology for bioinorganic speciation analysis: Metallomics, metalloproteomics and heteroatom-tagged proteomics and metabolomics. Analyst.

[CR57] Vazquez S, Carpena-Ruiz RO (2005). Use of perlite in cadmium plant studies: An approach to polluted soil conditions. Journal of Environmental Monitoring.

[CR58] Verbruggen N, Hermans C, Schat H (2009). Molecular mechanisms of metal hyperaccumulation in plants. New Phytologist.

[CR59] Wierzbicka MH, Przedpełska E, Ruzik R, Ouerdane L, Połeć-Pawlak K, Jarosz M, Szpunar J, Szakiel A (2007). Comparison of the toxicity and distribution of cadmium and lead in plant cells. Protoplasma.

[CR60] Woolhouse HW, Lange OL, Nobel PS, Osmond CB, Ziegler H (1983). Toxicity and tolerance in the responses of plants to metals. Encyclopedia of plant physiology: Physiological plant ecology.

[CR61] Yanai J, Zhao FJ, McGrath SP, Kosaki T (2006). Effect of soil characteristics on Cd uptake by the hyperaccumulator *Thlaspi caerulescens*. Environmental Pollution.

[CR62] Yang XE, Long XX, Ye HB, He ZL, Stoffella PJ, Calvert DV (2004). Cadmium tolerance and hyperaccumulation in a new Zn-hyperaccumulating plant species (*Sedum alfredii* Hance). Plant and Soil.

[CR63] Zhang GP, Fukami M, Sekimoto H (2002). Influence of cadmium on mineral concentrations and yield components in wheat genotypes differing in Cd tolerance at seedling stage. Field Crops Research.

[CR64] Zhao FJ, Jiang RF, Dunham SJ, McGrath SP (2006). Cadmium uptake, translocation and tolerance in the hyperaccumulator *Arabidopsis halleri*. New Phytologist.

