# Supplementary material for: The Influence of Cadmium Stress on the Content of Mineral Nutrients and Metal-Binding Proteins in Arabidopsis halleri
Source: Water Air Soil Pollut. 2012 Aug 23;223(8):5445–58. doi: 10.1007/s11270-012-1292-4 (PMC3443489; doi:10.1007/s11270-012-1292-4)
Supplement: Supplementary file 1 — Scatter plots showing correlations between Cd and mineral nutrient content in A. halleri roots. Regression lines (solid line), 95 % confidence intervals (dotted lines), coefficient of determination (R 2), and the equation of the regression line were given for each element. a 114Cd vs. 112Cd, b 24 Mg vs. 112Cd, c 32 S vs. 112Cd, d 55Mn vs. 112Cd, e 58Fe vs. 112Cd, f 63Cu vs. 112Cd, g 66Zn vs. 112Cd, h 98Mo vs. 112Cd. Element concentrations were quantified using ICP-MS (PDF 93 kb) [file 11270_2012_1292_MOESM1_ESM.pdf]

attachment to manuscript
Click here to download attachment to manuscript: Supplementary data Fig ESM 1.pdf
Click here to view linked References

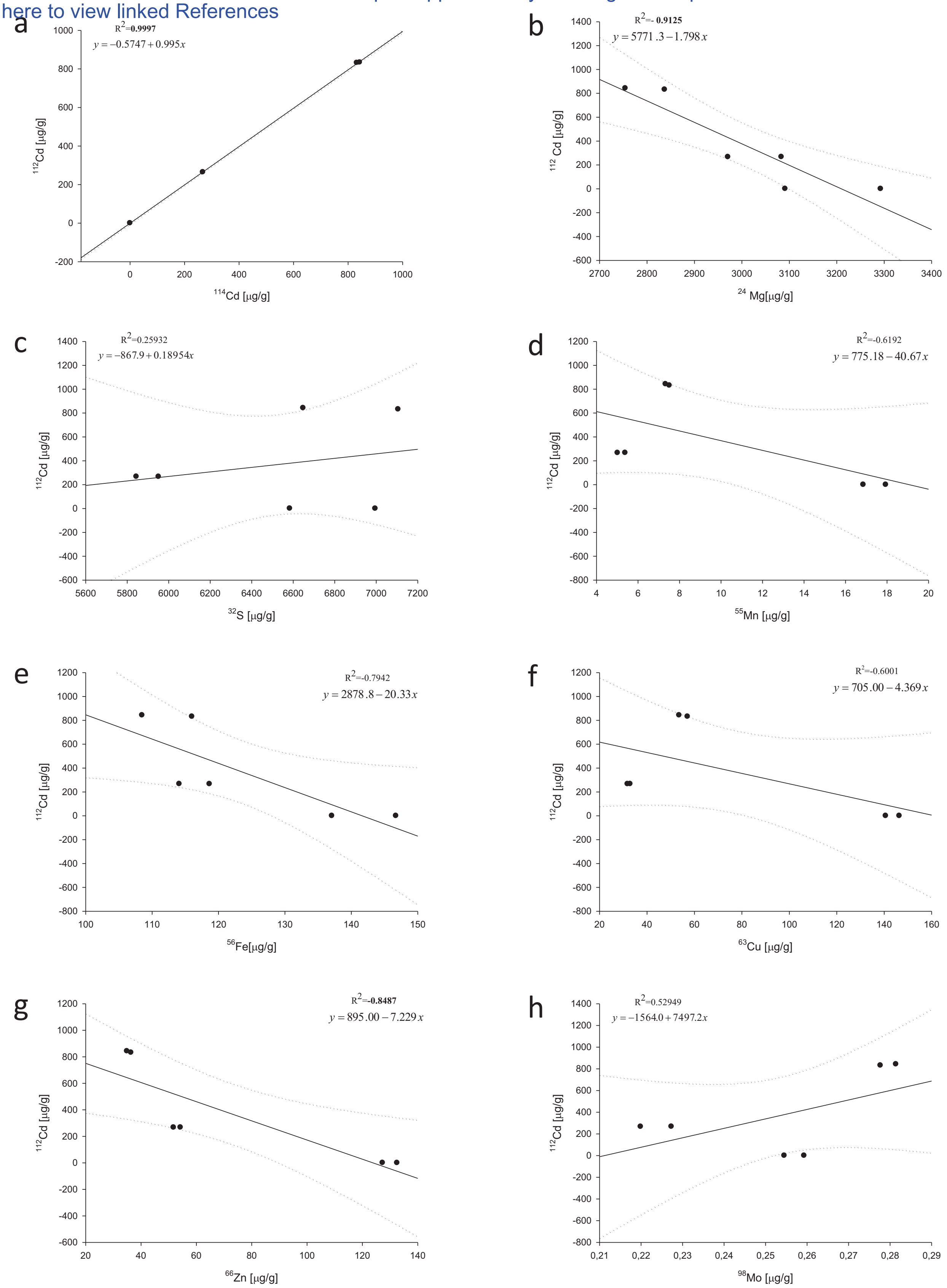


**Fig. ESM1 a-h** Scatter plots showing correlations between Cd and mineral nutrient content in *A. halleri* roots. Regression lines (solid line), 95 % confidence intervals (dotted lines), coefficient of determination ($R^2$) and the equation of the regression line were given for each element. (a) $^{114}Cd$ vs· $^{112}Cd$, (b) $^{24}Mg$ vs· $^{112}Cd$, (c) $^{32}S$ vs· $^{112}Cd$, (d) $^{55}Mn$ vs· $^{112}Cd$, (e) $^{58}Fe$ vs· $^{112}Cd$, (f)$^{63}Cu$ vs· $^{112}Cd$, (g) $^{66}Zn$ vs· $^{112}Cd$, (h) $^{98}Mo$ vs· $^{112}Cd$. Element concentrations were quantified using ICP-MS
