# Supplementary material for: The Influence of Cadmium Stress on the Content of Mineral Nutrients and Metal-Binding Proteins in Arabidopsis halleri
Source: Water Air Soil Pollut. 2012 Aug 23;223(8):5445–58. doi: 10.1007/s11270-012-1292-4 (PMC3443489; doi:10.1007/s11270-012-1292-4)
Supplement: Supplementary file 2 — Scatter plots showing correlations between Cd and mineral nutrient content in A. halleri shoots. Regression lines (solid line), 95 % confidence intervals (dotted lines), coefficient of determination (R 2), and the equation of the regression line were given for each element. a 114Cd vs. 112Cd, b 24 Mg vs. 112Cd, c 32 S vs. 112Cd, d 55Mn vs. 112Cd, e 58Fe vs. 112Cd, f 63Cu vs. 112Cd, g 66Zn vs. 112Cd, h 98Mo vs. 112Cd. Element concentrations were quantified using ICP-MS (PDF 104 kb) [file 11270_2012_1292_MOESM2_ESM.pdf]

attachment to manuscript
Click here to download attachment to manuscript: Supplementary data Fig ESM 2.pdf
Click here to view linked References

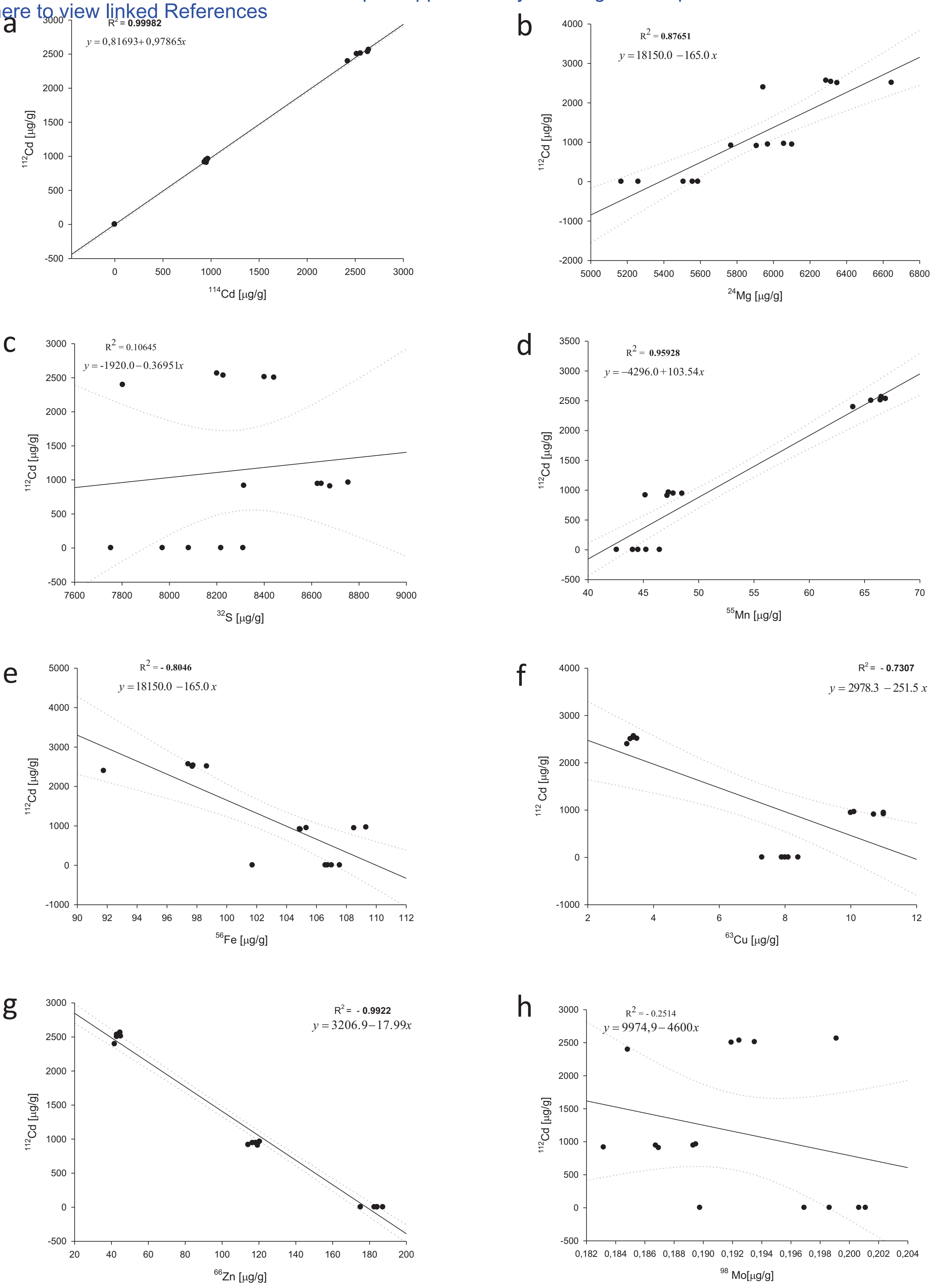

Fig. ESM2 a-h Scatter plots showing correlations between Cd and mineral nutrient content in *A. halleri* shoots. Regression lines (solid line), 95 % confidence intervals (dotted lines), coefficient of determination (R^2) and the equation of the regression line were given for each element. (a) ^{114}Cd vs ^{112}Cd, (b) ^{24}Mg vs ^{112}Cd, (c) ^{32}S vs ^{112}Cd, (d) ^{55}Mn vs ^{112}Cd, (e) ^{58}Fe vs ^{112}Cd, (f)^{63}Cu vs ^{112}Cd, (g) ^{66}Zn vs ^{112}Cd, (h) ^{98}Mo vs ^{112}Cd. Element concentrations were quantified using ICP-MS
